# Apigenin inhibits C5a-induced proliferation of human nasopharyngeal carcinoma cells through down-regulation of C5aR

**DOI:** 10.1042/BSR20180456

**Published:** 2018-05-15

**Authors:** Yanshu Zhang, Ying Cao, Linlin Zhang, Chunyan Feng, Guangquan Zhou, Guohua Wen

**Affiliations:** 1 Department of Ear-Nose-Throat, Yancheng First People’s Hospital, Fourth Affiliated Hospital of Nantong University, Yancheng 224001, P.R. China; 2 Department of Ear-Nose-Throat, The Second People’s Hospital of Huai’An, Huai’An Affiliated Hospital of Xuzhou Medical University, Huai’An 223002, P.R. China; 3 Department of Pharmacy, The First People’s Hospital of Yancheng, Fourth Affiliated Hospital of Nantong University, Yancheng 224001, P.R. China

**Keywords:** Apigenin, C5a, C5aR, nasopharyngeal carcinoma, proliferation

## Abstract

Complement 5a (C5a) is able to induce the proliferation of human nasopharyngeal carcinoma (NPC) cells. Therefore, an effective method or drug that can specifically inhibit C5a-induced proliferation of human NPC cells needs to be developed. Reportedly, Apigenin has antiproliferative effects on a variety of cancer cells. However, the effect of Apigenin on NPC cell proliferation and its underlying mechanism are still unclear. Herein, the present study aimed to evaluate the effect of Apigenin on C5a-induced proliferation of human NPC cells and its possible mechanism through down-regulation of C5aR. We revealed that Apigenin *in vitro* could not only inhibit proliferation of NPC cells and but also reduce the expression of C5aR and P300/CBP-associated factor (PCAF) as well as the activation of signal transducer and activator of transcription 3 (STAT3) in NPC cells. Furthermore, Apigenin reduced the proliferation of human NPC cells triggered by C5a through negative regulation of C5aR/PCAF/STAT3 axis. These might provide a new insight into the function of Apigenin in cancer treatment, and also provide a potential strategy for treating human NPC through inhibition of C5aR expression on cancer cells.

## Introduction

Nasopharyngeal carcinoma (NPC) is a common tumor in the head and neck arising from the epithelium of the nasopharynx [1,2]. It is one of the most common malignancies in Southern China and Southeast Asia with an incidence rate of 20–30 per 100000, accounting for approximately 40% of the world’s new cases [3–5]. Many studies have been done to explore the underlying mechanism of NPC [6,7]; however, the pathogenesis of NPC formation and development remains largely unclear.

Reportedly, complement activation contributes to the growth and metastasis tumors, such as gastric cancer, colon cancer, and lung cancer [8,9]. Some studies have revealed that the generation of complement components especially complement 5a (C5a) in the tumor microenvironment leads to significant tumor progression, such as lung cancer, gastric cancer, and renal cancer [10–12]. For example, Kaida et al. [11] reported that C5a receptor (CD88) could promote motility and invasiveness of gastric cancer by activating RhoA. Notably, Cai et al. [13] reported that C5a could promote the proliferation of human NPC cells through p300/CBP-associated factor (PCAF)-mediated acetylation of signal transducer and activator of transcription 3 (STAT3). Therefore, an effective method or drug that can inhibit C5a-induced proliferation of human NPC cells needs to be developed.

Apigenin, a natural plant flavonoid (4′,5,7-trihydroxyflavone), is widespread in common fruits and vegetables. Apigenin has been shown to have marked anti-inflammatory and antioxidant properties [14–17]. Recently, researchers have demonstrated that Apigenin has antiproliferative effects on a variety of cancer cells, such as lung, bladder, and breast cancers [18–20]. Additionally, Apigenin has been reported to regulate expression and/or activation of transcriptional factors, signaling molecules, and miRNAs, such as activation protein 1 (AP-1), NF-κB, extracellular signal-regulated kinase (ERK), p38 mitogen-activated protein kinase (MAPK), and *miR-155* [14,21–23]. However, the effects of Apigenin on NPC cells especially C5a-induced NPC cell proliferation are unclear.

In the present study, we set out to evaluate the effect of Apigenin on C5a-induced proliferation of human NPC cells and its potential mechanism through down-regulation of C5aR and inhibition of C5aR/PCAF/STAT3 axis.

## Materials and methods

### Reagents

Apigenin was purchased from Sigma-Aldrich (St. Louis, MO, U.S.A.). Monoclonal antibody against C5aR (sc-271949) was supplied by Santa Cruz Biotechnology (Dallas, TX, U.S.A.). Monoclonal antibodies against human PCAF (3378), STAT3 (9139), and β-actin (3700) were from Cell Signaling Technology (Danvers, MA, U.S.A.). Horseradish peroxidase-conjugated anti-mouse IgG (7076) and anti-rabbit IgG (7074), as well as ECL detection system were purchased from Cell Signaling Technology. PVDF membranes were from Millipore (Billerica, MA, U.S.A.). The pcDNA3.1 vector was from Invitrogen. The incision enzymes of HindIII and BamHI as well as T4 DNA ligase were purchased from TaKaRa (Tokyo, Japan). Human C5a was from R&D Systems (Minneapolis, MN, U.S.A.). Cell counting kit-8 (CCK-8) was purchased from Dojindo Laboratories (Kumamoto, Japan). FuGENE®HD was from Promega (Madison, WI, U.S.A.).

### Cell culture

The human NPC cell line of C666-1 was obtained from the American Type Culture Collection (ATCC, Manassas, U.S.A.). Cell lines were cultured in RPMI-1640 medium from Gibco (Carlsbad, CA, U.S.A.) supplemented with 10% FBS (Gibco) at 37°C in 5% CO_2_.

### Generation of overexpression plasmids

The plasmids of pcDNA3.1/C5aR and pcDNA3.1/STAT3 were constructed by inserting the ORF of human C5aR and STAT3 cDNA into pcDNA3.1, respectively. *C5aR* and *STAT3* genes were amplified by PCR from cDNA of normal human nasopharyngeal epithelial cells. The PCR products and pcDNA3.1 vector were further digested with the two restriction enzymes of HindIII and BamHI, and then ligated by using T4 DNA ligase. The plasmid of pcDNA3.1/PCAF-His was a gift from Dr Xueli Bao (Taizhou People’s Hospital, China).

### Cellular transfection

Cells were transfected with FuGENE®HD according to the manufacturer’s instructions. Briefly, cells were seeded in a six-well plate at 24 h before transfection (5 × 10^5^/well). Four micrograms of plasmids were mixed with 400 μl serum-free RPMI-1640 medium, and then 16 μl FuGENE®HD was added and incubated for 15 min at room temperature. Finally, the resultant mixture was added to cells in each well with 2 ml RPMI-1640 medium plus 10% FBS. The medium was replaced at 12 h after transfection.

### Immunoprecipitation

Three hundred and fifty microgram of extract prepared from cells was mixed with 40 μl protein G-Sepharose beads in co-immunoprecipitation (IP) assay buffer, incubated at 4°C for 3 h and centrifuged for 3 min. The supernatant was recovered and incubated with the corresponding antibody (2 μg, pre-immune IgG as a control reaction) at 4°C overnight. Then, 40 μl protein G-Sepharose beads were added into the tubes, and continued to be incubated at 4°C for 3 h. Protein G-precipitated protein complex was recovered by centrifugation and harvested beads resuspended in 50 μl SDS/PAGE sample buffer.

### Western blot analysis

The proteins (40 μg/well) were subjected to ExpressPlus™ PAGE Gel for electrophoresis (Genscript, Nanjing, China) and then transferred on to PVDF membranes. The PVDF membranes were incubated for 1 h at room temperature (RT) in blocking buffer (5% skim milk in TBS-T) and then incubated with the different antibodies overnight at 4°C. After washing with TBST-T for three times, the membranes were incubated with horseradish peroxidase-conjugated anti-rabbit and anti-mouse for 1 h at 37°C. The bands were visualized by the ECL detection system with 2–8 min exposure after washing the membranes. The radiographic band density was measured by using Quantity One software (Bio-Rad, Hercules, U.S.A.).

### CCK-8 assay

NPC cells after the different treatments were incubated with CCK-8 (1:10 dilution) for the final 2 h. The formazan product was detected at an absorbance of 450 nm, and the absorbance was directly proportional to the cell numbers [24].

### Statistical analysis

All statistical analyses were carried out by using SPSS 19.0 software. All data are given as mean ± S.D. One-way ANOVA with simultaneous multiple comparisons amongst groups by the Bonferroni method were performed, and the statistical significance was defined as *P*<0.05.

## Results

### Apigenin inhibits C5a-induced proliferation of human NPC cells

**Figure 1:**
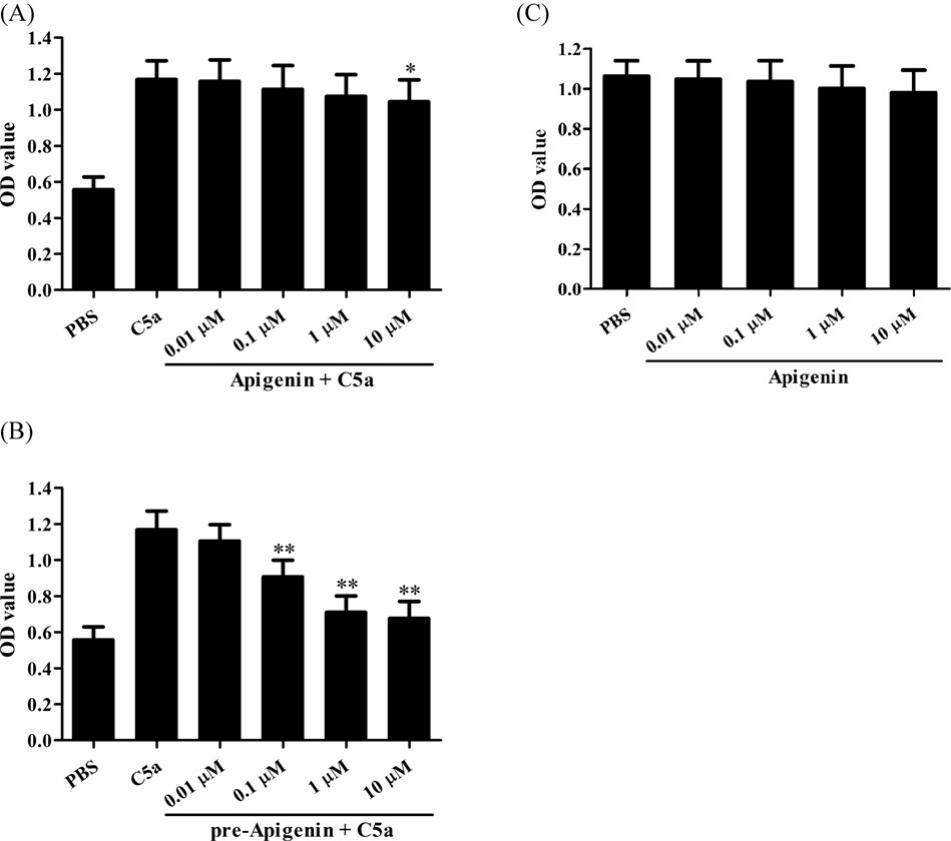
The effect of Apigenin on C5a-induced C666-1 cell proliferation (**A**) C666-1 cells were incubated with different doses of Apigenin (0.01, 0.1, 1, and 10 µM) and 20 ng/ml C5a for 48 h. CCK-8 assay was used to detect the proliferation levels of C666-1 cells in different groups. *P<0.05 compared with C5a group. (**B**) C666-1 cells were incubated with different doses of Apigenin (0.01, 0.1, 1, and 10 µM) for 24 h, and then stimulated with 20 ng/ml C5a for 48 h. CCK-8 assay was used to detect the proliferation levels of C666-1 cells in different groups. **P<0.01 compared with C5a group. (**C**) NP69 cells were incubated with 1 µM Apigenin for 48 h. CCK-8 assay was used to detect the proliferation levels of NP69 cells in different groups. Results were shown as means ± S.D. The data are from one experiment, representative of three independent experiments. Results were shown as means ± S.D.

To observe the effect of Apigenin on C5a-induced human NPC cell proliferation, the cultured C666-1 cells were treated with Apigenin at different doses (0.01, 0.1, 1, and 10 µM, respectively) and C5a at the dose of 20 ng/ml for 48 h. Cell proliferation was detected, and the result showed that Apigenin treatment could not significantly inhibit the proliferation of C666-1 cells induced by C5a (Figure 1A). Then, we further explore the effect of pretreatment with Apigenin on C5a-induced C666-1 proliferation. C666-1 cells were incubated with Apigenin at above-mentioned different doses for 24 h, followed by C5a stimulation at the dose of 20 ng/ml. After 48 h, cell proliferation was evaluated, and we observed that Apigenin pretreatment could markedly inhibit the proliferation of C666-1 cells induced by C5a in a dose-dependent manner (Figure 1B). These data suggest that Apigenin might inhibit NPC cell proliferation exposed to C5a possibly at the C5a-C5aR level. Apigenin could not significantly inhibit the proliferation of NP69 cells (Figure 1C).

### Apigenin inhibits C5a-induced proliferation of human NPC cells through down-regulation of C5aR

**Figure 2:**
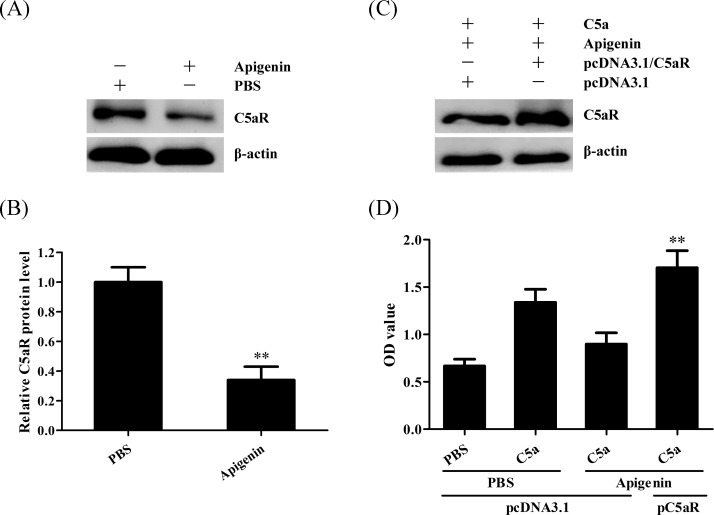
Apigenin inhibits C5a-induced proliferation of human NPC cells through down-regulation of C5aR (**A**,**B**) C666-1 cells were incubated with 1 µM Apigenin for 24 h, and then the expression of C5aR on C666-1 cells were detected by Western blot. **P<0.01 compared with PBS group. (**C**,**D**) C666-1 cells were transfected with pcDNA3.1/C5aR or pcDNA3.1 for 36 h, and then incubated with 1 µM Apigenin for 24 h, subsequently stimulated with 20 ng/ml C5a for 48 h. C5aR expression in the C666-1 cells at 24 h after Apigenin treatment was determined by Western blot (C) and cell proliferation at 48 h after C5a treatment was detected by CCK-8 (D). **P<0.01 compared with pcDNA3.1 + Apigenin + C5a group. The data are from one experiment, representative of three independent experiments. Results were represented as means ± S.D.

Since Apigenin could markedly inhibit the proliferation of C666-1 cells by exposure to C5a (Figure 1), we asked the question that whether Apigenin could inhibit C5a-induced proliferation of human NPC cells through down-regulation of C5aR. We first detected the expression of C5aR on C666-1 cells, and we found that Apigenin could obviously reduce the expression of C5aR on C666-1 cells (Figure 2A,B). In order to confirm the role of Apigenin-induced C5aR down-regulation in NPC cell proliferation, we performed overexpression experiment of C5aR. The results showed that C5aR overexpression (Figure 2C) could reverse the effect of Apigenin on C666-1 proliferation in response to C5a (Figure 2D). Taken together, these data indicate that Apigenin inhibits C5a-induced proliferation of human NPC cells through down-regulation of C5aR.

### PCAF expression is involved in Apigenin-reduced C5a-induced proliferation of human NPC cells

**Figure 3:**
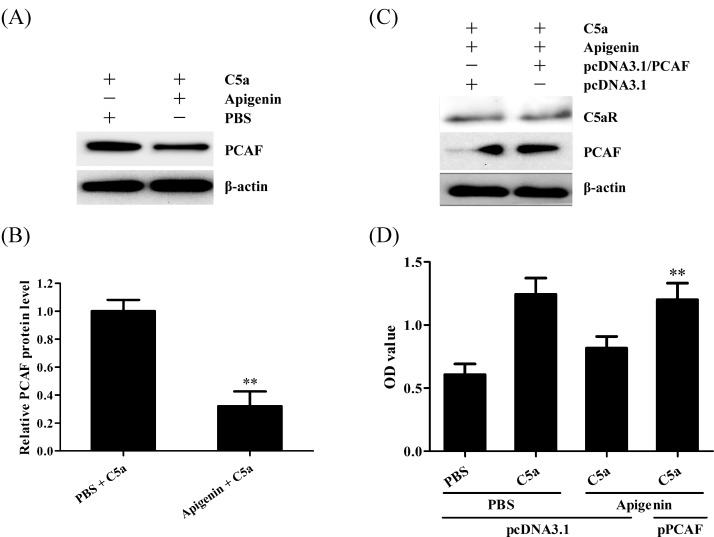
PCAF expression is involved in Apigenin-reduced C5a-induced proliferation of human NPC cells (**A**,**B**) C666-1 cells were incubated with 1 µM Apigenin for 24 h followed by 20 ng/ml C5a stimulation for 3 h, and then the expression of PCAF in the C666-1 cells were detected by Western blot. **P<0.01 compared with PBS group. (**C**,**D**) C666-1 cells were transfected with pcDNA3.1/PCAF or pcDNA3.1 for 36 h, and then incubated with 1 µM Apigenin for 24 h, subsequently treated with 20 ng/ml C5a for 48 h. The expression levels of C5aR and PCAF in the C666-1 cells at 3 h after C5a treatment were determined by Western blot (C) and cell proliferation at 48 h after C5a treatment was detected by CCK-8 (D). **P<0.01 compared with pcDNA3.1 + Apigenin + C5a group. The data are from one experiment, representative of three independent experiments. Results were shown as means ± S.D.

PCAF expression is involved in C5a-induced NPC cell proliferation [13], therefore, we further checked the effect of Apigenin on PCAF expression in NPC cells. The results showed that Apigenin treatment could markedly inhibit PCAF expression in the C666-1 cells stimulated with C5a (Figure 3A,B). Functionally, PCAF overexpression could reduce the effect of Apigenin on C666-1 proliferation (Figure 3D), but had no effect on C5aR expression in Apigenin-treated C666-1 cells (Figure 3C), confirming the upstream and downstream relationship between C5aR down-regulation and PCAF down-regulation. These data indicate that Apigenin reduces the proliferation of human NPC cells triggered by C5a through negative regulation of PCAF.

### STAT3 acetylation is related to Apigenin-reduced C5a-induced proliferation of human NPC cells

**Figure 4:**
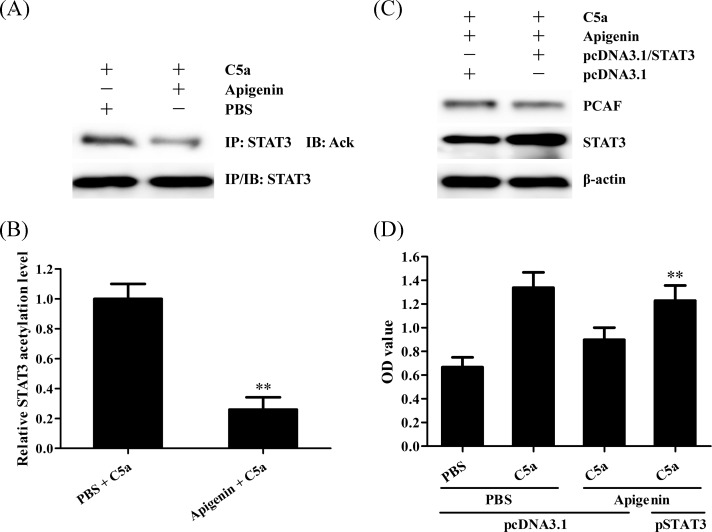
STAT3 acetylation is related to Apigenin-reduced C5a-induced proliferation of human NPC cells (**A**,**B**) C666-1 cells were incubated with 1 µM Apigenin for 24 h followed by 20 ng/ml C5a stimulation for 3 h, and then the acetylation levels of STAT3 in the C666-1 cells were detected by IP assay. **P<0.01 compared with PBS group. (**C**,**D**) C666-1 cells were transfected with pcDNA3.1/STAT3 or pcDNA3.1 for 36 h, and then incubated with 1 µM Apigenin for 24 h, subsequently stimulated with 20 ng/ml C5a for 48 h. The expression levels of PCAF and STAT3 in the C666-1 cells at 3 h after C5a stimulation was determined by Western blot (C) and cell proliferation at 48 h after C5a stimulation was detected by CCK-8 (D). **P<0.01 compared with pcDNA3.1 + Apigenin + C5a group. The data are from one experiment, representative of three independent experiments. Results were represented as means ± S.D.

Given that STAT3 acetylation is involved in C5a-induced NPC cell proliferation, we also checked the acetylation of STAT3 in NPC cells treated with Apigenin. The results showed that Apigenin treatment could obviously inhibit STAT3 acetylation in C666-1 cells induced by C5a (Figure 4A,B). Furthermore, STAT3 overexpression could reduce the effect of Apigenin on C666-1 proliferation in response to C5a (Figure 4D), but had no effect on PCAF expression in Apigenin-treated C666-1 cells (Figure 4C), confirming the upstream and downstream relationship between PCAF down-regulation and STAT3 inactivation induced by Apigenin. Taken together, these data indicate that Apigenin reduces the proliferation of human NPC cells triggered by C5a through negative regulation of C5aR/PCAF/STAT3.

## Discussion

NPC is a common tumor in the head and neck in clinic especially in Asia; however, the molecular pathogenesis of NPC remains largely unclear. Recently, complement activation especially C5a generation is believed to contribute to cancer progression [11,12,25]. Cai et al. [13] reported that C5a could promote the proliferation of human NPC cells through PCAF up-regulation and PCAF-mediated acetylation of STAT3. Therefore, an effective method or drug that can inhibit C5aR, PCAF, and STAT3 expression and activation in NPC cells needs to be developed.

Apigenin, a natural plant flavonoid (4′,5,7-trihydroxyflavone), is widespread in common fruits and vegetables. Apigenin has been shown to have marked antiproliferative effects on a variety of cancer cells, such as lung, bladder, and breast cancers [18–20]. However, the effects of Apigenin on NPC cells especially C5a-induced NPC cell proliferation are unclear. Our current study for the first time revealed that Apigenin pretreatment could inhibit proliferation of NPC cells induced by C5a; however Apigenin treatment at the same time with C5a treatment had no significant effect. These data indicate that Apigenin might affect C5a-induced C666-1 cell proliferation through the regulation of C5aR expression. Therefore, the effect of Apigenin on C5aR expression was further explored. Expectedly, we observed that Apigenin could reduce the expression of C5aR on NPC cells. We further proposed a hypothesis that Apigenin might inhibit C5a-induced proliferation of human NPC cells through the inhibition of C5aR/PCAF/STAT3 axis. In the following studies, we set out to evaluate the potential mechanism of Apigenin on NPC cells. We found that Apigenin reduces the proliferation of human NPC cells triggered by C5a through negative regulation of C5aR/PCAF/STAT3 axis, amongst which reduction in C5aR is the key mechanism.

It is worthy to mention that Apigenin is different from traditional C5 inhibitor or neutralizing antibodies such as eculizumab [26]. Eculizumab can not only reduce the level of C5a *in vivo*, but also inhibit the formation of C5b-9. In contrast, Apigenin exhibits more specific effect via the reduction in C5aR on NPC cells. This might exhibit stronger effects on cancer cells with high expression of C5aR. In addition, it has been reported that Apigenin is able to regulate expression and/or activation of some transcriptional factors, signaling molecules, and miRNAs in target cells [14,21–23]. Further studies need to be done to explore whether Apigenin could reduce C5aR expression in NPC cells through the regulation of above-mentioned molecules.

## Abbreviations

BamH-I: Bacillus amyloliquefaciens H-I
CCK-8: cell counting kit-8
C5a: complement 5a
c5b9: terminal complement comples (TCC) c5b-9
c5aR: c5a receptor
CBP: CREB-binding protein
CD88: cluster of differentiation 88
C666-1: C666-1 nasopharyngeal carcinoma cells
Hind III: *Haemophilus influenzae* deoxyribonuclease III
NF-κB: nuclear factor kappa-light-chain-enhancer of activated B cells
NPC: nasopharyngeal carcinoma
NP69: nasopharyngeal carcinoma cell 69
PCAF: p300/CBP-associated factor
p300-CBP: p300-CREB-binding protein
STAT3: signal transducer and activator of transcription 3
TBS-T: triethanolamine buffered saline solution-tween
